# Synergism of arbuscular mycorrhizal fungi and bacteria in bioremediation and restoration of metal-stressed environments

**DOI:** 10.3389/fpls.2026.1753034

**Published:** 2026-02-20

**Authors:** Hamid Amir, Linda Guentas, Thomas Crossay, Alexandre Bourles, Valérie Burtet-Sarramegna

**Affiliations:** 1 Institut des Sciences Exactes et Appliquées (ISEA), Université de la Nouvelle-Calédonie, Noumea, New Caledonia; 2 AURA-PACIFICA Company, Noumea, New Caledonia

**Keywords:** arbuscular mycorrhizal fungi, metal pollution, mycorrhiza helper bacteria, phytoremediation, plant growth-promoting rhizobacteria, restoration, synergistic effects

## Abstract

Metal pollution poses significant ecological and economic concerns for many countries, resulting from anthropogenic activities such as intensive farming, mining, and other industrial sectors. Many of these metals can be toxic, affecting not only plant and animal nutrition but also human health. Phytoremediation of metal-polluted soils is now regarded as one of the most promising nature-based solutions for removing metals from contaminated environments. It can be enhanced by plant inoculation with beneficial microorganisms, such as arbuscular mycorrhizal fungi (AMF) and plant growth-promoting rhizobacteria (PGPR). For about two decades, the combined use of PGPR and AMF has attracted interest. This review summarizes the studies carried out on this subject, highlighting the complementary mechanisms of these two types of microbes and their synergistic effects, which improve the plant’s mineral nutrition and tolerance to heavy metals, as well as better metal neutralization through stabilization in the plant’s aerial and root organs and in the soil. Among these mechanisms, AMF intervene by mobilizing essential minerals due to their external mycelium, which explores a large volume of soil. AMF also contribute to reducing soil erosion through the soil-binding capacity of their extraradical mycelium and glomalin production, which enhances soil aggregation and stability. These symbionts contribute efficiently to metal toxicity alleviation in plants. PGPR can improve plant growth through various mechanisms, including hormone production, 1-aminocyclopropane-1-carboxylate (ACC) deaminase activity, nitrogen fixation, and the secretion of different chelating substances. Metals can be neutralized by a variety of processes, including binding, biosorption, transformation, and immobilization. Mycorrhiza helper bacteria are associated with AMF and can stimulate their mycelial growth, spore production, and spore germination, thus increasing mycorrhizal colonization. The selection of bacteria and AMF for phytoremediation purposes should be based on these different complementary properties. Furthermore, genomic and transcriptomic studies may be utilized to identify the most active genes in terms of their positive effects on the plant and phytoremediation mechanisms. This approach enables a more rigorous selection of strains. Field experiments with co-inoculation of AMF and bacteria are rare at present and need to be developed in different edaphic and climatic conditions.

## 1 Introduction

Metal contamination due to different industrial activities and transfer of metal rich particles from mines by surface erosion represents one of the most critical environmental concerns over the world (Alloway, 2013). Urban and agricultural sources of metal pollution also contribute to increased toxic metal concentrations in soil, including traffic exhaust, burning fuel, food waste, municipal sludge, agricultural practices (fertilizers, pesticides), electronics, and other commodity impurities, which can be spread in soils by rain and water runoff (Havugimana et al., 2017; Zwolak et al., 2019). Among toxic metals concerned by pollution, Mn, Cu, Co, Zn, Cr, and Ni are essential to plant metabolism at trace levels (Nagajyoti et al., 2010). However, their bioavailable concentrations can sometimes be sufficiently high to threaten soil functions. Others have no metabolic role, such as Pb, Cd, Hg, and As (Nagajyoti et al., 2010; Clemens and Ma, 2016). All these metals are dispersed by anthropogenic activities and can become toxic at concentrations varying with metal species and its chemical form, affecting plant and animal nutrition, but also human health (Alloway, 2013; Briand et al., 2018). Metal toxicity can also be an inherent trait of soil composition, in environments with metal rich rocks such as ultramafic areas (Proctor, 2003; Jaffré and L’huillier, 2010). Ultramafic soils are then naturally rich in potentially toxic metals such as Ni, Co, Cr, and Mn, in addition to main elements deficiencies (particularly P and K). Mine exploitation of these areas induces ecosystem degradation needing restoration.

The bioremediation of metal-polluted soils and the restoration of mine degraded lands require similar approaches, due to the soil’s infertility and potential toxicity of metals in both environments. If the remediation of brownfields is an important issue, particularly for their reuse in agriculture, the ecological restoration of mined areas, which cover large surfaces, is necessary, especially when they contain valuable biodiversity and need to reduce metal toxicity. Research in this field began mainly in the nineties (Dong et al., 2024) and has become an important issue in recent years, particularly in the fight against climate change (Aronson and Alexander, 2013; Fischer et al., 2021). To achieve this objective, ecological approaches have been constantly improving over the past two decades. These approaches aim to maintain as much as possible the local biodiversity, which requires the rehabilitation of soil functions, including optimization of microbiological balances (Williams et al., 2024).

**Figure 1:**
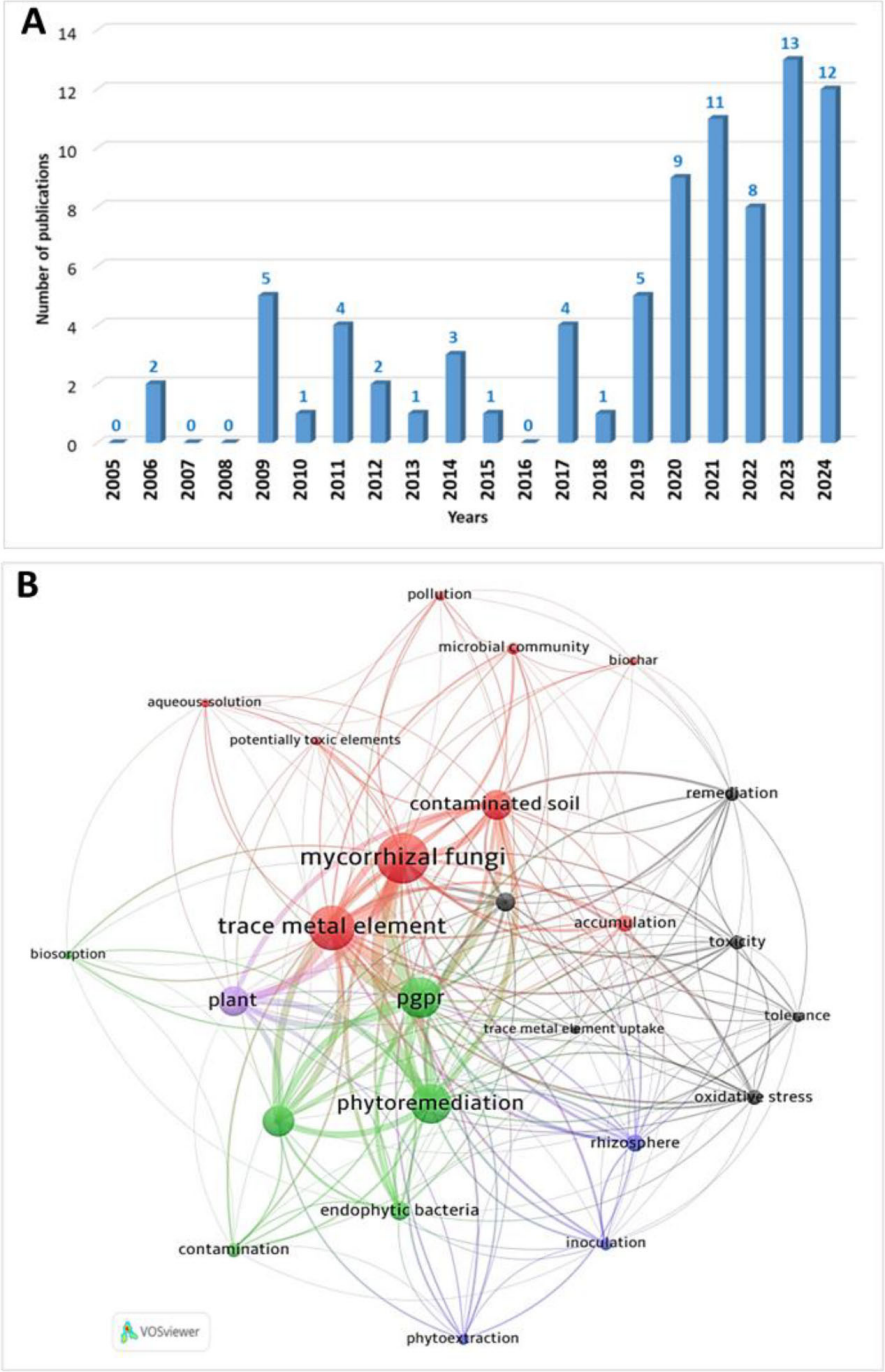
**(A)** Numbers of publications dealing with combined effects of AMF and bacteria in phytoremediation and restoration of metal-stressed Soils, over the last 20 years. **(B)** Keyword co-occurrence map showing the most frequently investigated topics.

Indeed, a significant part of soil microbiota interacts positively with plants for their development. This is particularly the case in stressed conditions such as nutrient deficiency, water stress, and metal toxicity (Augé et al., 2015; Bourles et al., 2020; Raklami et al., 2022b; Hnini et al., 2024). Thus, using microorganisms in terrestrial ecosystem restoration and soil bioremediation perspectives is increasingly considered relevant. Microbial interactions have important implications in ecosystem conservation and restoration, particularly for the alleviation of different soil stresses such as salinity, drought, and heavy metals (Williams et al., 2024). According to Gupta et al. (2024), the use of microorganisms to treat heavy metal contamination of soils is an ecofriendly and sustainable method. A search in Web of Science with the keywords “bacteria” or “AMF” (arbuscular mycorrhizal fungi) and “metals”, and “restoration”or “phytoremediation” showed 225 publications for the decade 2005–2014 and 601 for the decade 2015-2024. For the keywords “bacteria” and “AMF” with the same other key words, we obtained only 82 publications (**Figure 1A**), part of them corresponding to review articles about phytoremediation of metal polluted soils using microorganisms, including co-inoculations. Among these 82 publications, only 18 were published during the decade 2005-2014, and 64 during the decade 2015-2024, 53 being published in the last five years. These figures clearly show that research interest in co-inoculating bacteria and AMF for phytoremediation and soil restoration is very recent. The relative importance of the different concepts related to the subject and their interactions are shown in **Figure 1B**.

AMF are ubiquitous obligate symbionts (Glomeromycota) associated with 90% of the angiosperms (Lanfranco et al., 2017). They are largely known to play different roles in plant growth and adaptation, including the alleviation of various abiotic stresses (Smith et al., 2010; Amir et al., 2014; Ferrol et al., 2016; Banerjee et al., 2025). Thus, the effects of these symbionts are of significance in the restoration or the remediation of degraded or polluted environments (Amir and Crossay, 2024; Hnini et al., 2024; Zhao et al., 2024).

Plant growth-promoting rhizobacteria (PGPR) correspond to rhizosphere or endophytic bacteria that exhibit the ability to stimulate plant growth (Bhattacharyya and Jha, 2012). They have also been largely investigated in restoration and bioremediation targets (Lies et al., 2018; Xiao et al., 2023; Hnini et al., 2024). They are particularly known for their metal biosorption and accumulation capabilities (Raklami et al., 2022b), thus mitigating toxicity to the plant. They can also stimulate plant growth through different mechanisms *(*Berg, 2009*;* Desai et al., 2016*;* Dong et al., 2024).

Mycorrhiza helper bacteria (MHB), belonging mainly to the Firmicutes, can interact with mycorrhizal fungi and promote the establishment of mycorrhizal symbiosis by stimulating root colonization, as well as seed germination and growth of their host (Lies et al., 2018; Nasslahsen et al., 2022).

Research attention on bacterial and fungal interactions to improve soil functions has been constantly increasing in recent years (Williams et al., 2024). According to Hnini et al. (2024), “In the context of phytoremediation of heavy metal-contaminated soils, the partnership between PGPR and mycorrhizal fungi, particularly AMF, emerges as a critical factor”. This review reports the use of AMF and bacteria, focusing particularly on their complementarity for the treatment of metal pollutions and the enhancement of plant growth and stress tolerance in soil remediation and ecosystem restoration.

## 2 Use of AMF and PGPR in ecological restoration and bioremediation

Due to the potential toxicity of metals in both environments, and the resulting infertility, the remediation of metal-contaminated soils and mine degraded lands needs deep scientific knowledge. In these conditions, research on microbial strains that improve the plant’s growth and adaptation to multiple stresses occurring in these media is particularly appropriate (Rajkumar et al., 2009; Amir and Crossay, 2024).

### 2.1 AMF

Among fungi, AMF present the advantage of having a very close physiological relationship with the plant. They are ubiquitous and concern the majority of vascular plants. Moreover, these fungi are characterized by low specificity (Sanders, 2002; Smith and Read, 2008), so that the same strain can be used for many plant species. According to Zhang X. et al. (2024), “the symbiosis between AMF and plants is considered to be one of the most effective ways to counteract biotic and abiotic stresses, including remediation of contaminated media and enhancement of plant tolerance”. Research in this field began to be significant in the nineties (Shetty et al., 1994; Leyval et al., 1997) and has been more and more developed from the 2000s onwards (Amir et al., 2014; Valliere et al., 2020; Zhang X. et al., 2024).

AMF are frequent in harsh conditions, such as highly metal-polluted soils and naturally metal-rich soils (Vallino et al., 2006; Hassan et al., 2010; Amir et al., 2014; Wang, 2017), and can be particularly efficient in conditions of abiotic and biotic stresses (Amir and Crossay, 2024; Zhang X. et al., 2024). Their beneficial effects on plants occur mainly through an extensive mycelium that explores a broad volume of soil, improving plants’ ability to exploit the substrate, then increasing plant nutrition (Marschner and Dell, 1994; Smith and Read, 2008; Khalid et al., 2021). Thus, mycorrhizal symbiosis can improve the uptake of several mineral nutrients. One of the most significant outcomes of AMF inoculation is the enhancement of P absorption (Marschner, 1995; Smith and Read, 2008); yet this element is generally characterized by low availability, being frequently bound to clay minerals (Smith and Read, 2008). In naturally metal-rich soils, P can be rare or weakly available because of its adsorption in metal complexes (Jaffré and L’huillier, 2010). AMF-inoculated plants also frequently absorb more K, N, and micronutrients (Hassan et al., 2010; Crossay et al., 2019; Zhao et al., 2024). In ultramafic soils, where Ca/Mg ratio is low, inducing Ca unavailability, AMF increase this ratio, leading to better Ca absorption (Amir et al., 2019). AMF can also contribute to reducing soil erosion, due to soil binding capacity of extraradical mycelium (Mozafar et al., 2002; Vosátka et al., 2012) and glomalin production (Rillig, 2004; Vodnik et al., 2008, Ahammed et al., 2023), which enhance soil aggregation and stability (Tisdall, 1994; Barea et al., 2005; Holátko et al., 2021).

Many AMF taxa have been found in heavy-metal-polluted and naturally metal-rich soils, among them, different species of *Glomus, Rhizophagus, Sclerocystis, Claroideoglomus, Acaulospora, Entrophospora, Scutellospora, Gigaspora*, and *Diversispora* (Khade and Adholeya, 2007; Amir et al., 2014; Wang, 2017; Amir et al., 2023). New species have also been described, particularly in ultramafic soils (Crossay et al., 2018, 2024). These symbionts have developed metal tolerance in metal-polluted soils (Leyval et al., 1997; Tullio et al., 2003; Hildebrandt et al., 2007). Wu et al. (2010) reported that spore germination of *Glomus mosseae* isolate from heavy metal-contaminated soils tolerated up to 15.5 μg g^-1^ Pb. Isolates of *Claroideoglomus etunicatum* from ultramafic soils can grow up to 30 μg g^−1^ Ni, whereas non-ultramafic isolates support only concentrations up to 10 μg g^−1^ Ni (Amir et al., 2008; Amir et al., 2013). However, colonization can be reduced by high concentrations of heavy metals in soil (Lingua et al., 2008; Amir et al., 2007; Gamalero et al., 2009). AMF contribute efficiently to metal alleviation in plants (Leyval et al., 2002; Amir et al., 2019; Crossay et al., 2019; Zhang X. et al., 2024). Field experiments have demonstrated that AMF can efficiently improve growth and adaptation of plants in ultramafic soils (Amir et al., 2019) and metal-polluted lands (Akhtar et al., 2020). Different mechanisms are involved in AMF’s ability to alleviate metal toxicity. These symbionts can use extracellular chelation, cell wall binding, metal accumulation in extraradical mycelium, and sequestration in the rhizosphere (Colpaert et al., 2011; Banerjee et al., 2025). Glomalin production contributes to metal chelation in soil (Gamalero et al., 2009; Banerjee et al., 2025). AMF can also neutralize metals using various molecules, such as organic acids, amino acids, glutathione, phytochelatins, and metallothioneins, and accumulate them in vacuoles (Amir et al., 2014). They also contribute to reducing oxidative stress due to metal toxicity through nonenzymatic antioxidant systems, such as glutathione, and enzymatic systems, such as catalase and superoxide dismutase (Ferrol et al., 2009; Neagoe et al., 2013). For all these properties, the use of AMF was proposed to improve restoration by increasing ecosystem productivity and stability (Dietrich et al., 2023). Also, according to Zhao et al. (2024), AMF-assisted phytoremediation is a promising strategy for Cd-contaminated soils. Mycorrhizal fungi can improve not only growth and yield of pot marigold in heavy metal stressed condition, but also phytoremediation performance by increasing heavy metals’ accumulation in plant organs (Tabrizi et al., 2015).

However, in practice, several difficulties have been outlined (Berruti et al., 2016; Hart et al., 2018; Zhao et al., 2024; Williams et al., 2024). In particular, the efficiency of AMF varies depending on edaphic conditions (Vosátka et al., 2012; Amir and Crossay, 2024). It is particularly the case of soil pH, affecting metal availability, and soil P concentration which affect mycorrhizal activity (Sivakumar, 2013; Kuyper and Jansa, 2023). Also, the mastering of AMF production and inoculation at large scale is still challenging and needs more research (Hart et al., 2018; Amir and Crossay, 2024).

### 2.2 PGPR

Bacteria constitute the most abundant group of microorganisms in the rhizosphere (Prashar et al., 2014). The structure of rhizospheric microbial communities can vary largely depending on plant species, particularly in relation to metal dynamics (Dijoux et al., 2025). Many rhizosphere bacteria have developed synergistic interactions with plants, these latter producing - through rhizodeposition - the carbon compounds and other nutrients necessary for bacterial growth (Hinsinger et al., 2009; Lagos et al., 2015). In turn, bacteria contribute to the release of mineral elements for plant growth, particularly through the degradation of organic matter (Hinsinger et al., 2009; Lagos et al., 2015). They can also stimulate plant growth through various mechanisms, characteristic of PGPR.

The utilization of PGPR as biofertilizers in stress-prone environments is gaining more and more interest (Hnini et al., 2024). According to Deb et al. (2020), the role of rhizosphere bacteria in phytoremediation can be direct, by stimulating metal uptake and translocation, thus enhancing the phytoextraction process. In other cases, they reduce metal mobility and availability within the rhizosphere, favoring phytostabilization. This role can also be indirect by improving plant tolerance to metals (Manoj et al., 2020; Akhtar et al., 2021) and stimulating plant growth, thus increasing the volume of tissues sequestrating or stabilizing metals (Dong et al., 2024). PGPR influence plant growth through different mechanisms such as hormones and 1-aminocyclopropane-1-carboxylate (ACC) deaminase production, nitrogen fixation, siderophore synthesis, and secretion of different other chelating substances (Gamalero et al., 2009; Dong et al., 2024; Hnini et al., 2024). Indoleacetic acid (IAA) production and nitrogen fixation are well known mechanisms of plant stimulation (Berg, 2009; Desai et al., 2016). ACC deaminase production minimizes ethylene secretion, thus positively affecting plant growth (Rajkumar et al., 2009; Sharma, 2021). Siderophore production by bacteria in the rhizosphere can sequestrate iron, thus allowing its uptake by plant (Ma et al., 2011), but can also contribute to neutralizing toxic metals, such as Ni, Pb, and Zn (Gamalero et al., 2009). Other chelating substances secreted in the rhizosphere can play important roles. Bourles et al. (2024) studied the plant growth-promoting effect of an exopolysaccharide (EPS) produced by *Paraburkholderia ultramafica*, a new species isolated from New Caledonian ultramafic soil. The EPS increased the growth of *Tetraria comosa*, a Cyperaceae endemic to New Caledonia, and reduced the translocation of Co, Cr, and Fe by chelation. PGPR can also contribute to the solubilization of phosphates, favoring their absorption by the root system (Ryan et al., 2008; Dong et al., 2024), which can also increase toxic metal extraction (Jeong et al., 2013). The role of phosphate solubilizing bacteria could be particularly important in ultramafic soils, generally characterized by P deficiency (Jaffré and L’huillier, 2010), as it was reported by Rajkumar and Freitas (2008) concerning strains of *Pseudomonas* isolated from ultramafic soils in Portugal. The role in P solubilization of an EPS produced by *P. ultramafica* has been highlighted by Bourles et al. (2024). PGPR can also contribute to maintaining soil structure and manage diseases and pests (Gupta et al., 2024).

The effects of PGPR on plants vary largely depending on different properties, so that a careful screening of the strains for the phytoremediation target is important, particularly knowing that they can increase or reduce the metal translocation factor (TF), with implications on phytoremediation potential (Akhtar et al., 2018). As for AMF, some difficulties are still frequent in the use of PGPR in practice, generally due to the same limits, in particular soil’s diversity inducing problems in maintaining the inoculants (Hart et al., 2018; Aloo et al., 2022). These limits are one of the main reasons to complexify the practices, with inoculants containing different groups of efficient microorganisms (Amir and Crossay, 2024). According to Aloo et al. (2022), “it is expected that the identification of effective microbiomes in different soil types and climates will be extremely helpful”.

## 3 Synergistic effects of AMF and bacteria in ecological restoration and bioremediation

### 3.1 AMF and PGPR

According to Williams et al. (2024), “promoting synergistic interactions between mycorrhizal fungi and soil microbes holds immense potential for advancing ecological knowledge and conservation”. The ecological niches of bacteria and AMF in the ecosystem are generally different, so that they do not enter in competition after their simultaneous inoculation to remediate soil perturbation. Even more, several mechanisms of their effects are also different, enabling complementarity in promoting plant health, particularly for the alleviation of soil stresses, including nutrition deficiencies and heavy metal toxicity.

AMF and PGPR complementarity amplify their collective effects on plant health and growth (Diagne et al., 2020). The study of Barzegari Barogh et al. (2023) is of particular interest to illustrate this complementarity. They tested the influence of AMF (*Rhizophagus intraradices* and *Funneliformis mosseae* strains) and PGPR (*Azotobacter chroococcum* and *Pseudomonas putida* strains) inoculated together or separately on potato plants cultivated in sterilized soil. These AMF strains proved a close symbiosis with plants through providing water and fundamental nutrients, *A. chroococcum* was characterized by N_2_-fixing activity, synthesis of siderophores, and indole-3-aceticacid stand, whereas *P. putida* could solubilize phosphates and produce auxins. The results showed that both the AMF and bacteria strains had a significant positive effect on potato plant growth and health, but their co-inoculation induced clearly higher effects. For the combination *of F. intraradices* and *P. putida*, shoot and root dry weights were significantly increased by 92.0% and 29.8%, respectively, and minituber weight was increased by 142.3% compared with the AMF treatment alone. This latter has already increased these parameters by more than 80% in comparison with non-inoculated plants. P and K uptake were also significantly improved with the *F. mosseae* and *P. putida* combination by 36.1% and 20.4%, respectively; whereas N uptake was significantly increased by the association of *F. mosseae* and *A. chroococcum* by 14.9%, all compared to the AMF treatment. The principal component analysis revealed that AMF and PGPR were positioned differently on the axes, and the heat map revealed different traits of the two treatments, confirming their influence at different levels of the plant’s physiology. Synergistic interactions between AMF and rhizobia have been reported to simultaneously induce an improvement of nodulation mediated by phosphate absorption, and AMF colonization, with consequential benefit to plant growth (Desai et al., 2016). The positive interaction between the AMF *Glomus fasciculatum* and the non-symbiotic bacteria *Azotobacter chroococum* was found to enhance plant growth (Desai et al., 2016). In this interaction, the mycorrhizal infection stimulated *A. chroococum* population in the rhizosphere, and the bacteria reciprocally enhanced the AMF spore production and root colonization. Bagyaraj et al. (2015) also found a synergistic interaction between AMF and phosphate-solubilizing bacteria inducing plant growth improvement. Dual inoculation of tomato seedlings with *Glomus bagyarajii* and PGPR *Methylocaterium radiotolerans* (Ranjitha et al., 2024) showed significant increase of plant dry weight (33.3% more than the AMF treatment alone), mycorrhizal colonization (10% more), and rhizosphere microbial populations.

This synergism also operate in metal neutralization, and metal stress alleviation in plant, as illustrated by Hnini et al. (2024) in a synthetic figure showing all the biological strategies used by the two collaborative microbes. Among these mechanisms, the authors cited production of metal chelators, metal biosorption and binding, metal sequestration and immobilization, metal transformation and reduction, metal uptake competition, detoxification enzyme induction, and enhanced plant tolerance. **Table 1** lists the main studies on the combined effects of AMF and bacteria aiming at the phytoremediation or the restoration of metal-stressed soils. To avoid repetitions, only some of them are commented on here. The study of Mokarram-Kashtiban et al. (2019) is particularly interesting in terms of synergistic effects between AMF and PGPR. The authors studied the influence of the inoculation by *Rhizophagus irregularis* and *Pseudomonas fluorescens* isolates on the metal phytoremediation ability of white willow (*Salix Alba*), in presence of nano-sized zero valent iron. The combination AMF-PGPR increased plant growth as well as physiological and metal uptake parameters. Total dry mass and leaf dry mass were improved by 23.6% and 22.7% respectively, compared to the AMF-only treatment. Chlorophyll a and b concentrations, net photosynthesis, stomatal conductance, and water-use efficiency were enhanced. Mycorrhizal colonization by *R. irregularis* increased in presence of the PGPR inoculum by 32.9%. Pb, Cu, and Cd concentrations in plant organs were higher in AMF-PGPR treatment by 20% to 150%, depending on organ and metal, in comparison with AMF-only treatment. Bioconcentration factors for Pb, Cu, and Cd were significantly higher by 39.5%, 29.2%, and 68.3%, respectively, compared with the AMF treatment. Vivas et al. (2003) inoculated white clover plants with a mix of Cd-tolerant bacteria and AMF, consisting of *Brevibacillus* sp. and *Glomus mosseae*, in soil contaminated with Cd. The mixed inoculant was more efficient than single ones for the improvement of shoot biomass (26% more than AMF single treatment in presence of 33 mg kg^-1^ Cd), phosphorus and nitrogen uptake, nodule number, mycorrhizal colonization (54% more), and reduction of Cd in plant shoots (37.5% less). Similar results were obtained with Zn (Vivas et al., 2006). Li et al. (2020) reported synergistic influence of *Funneliformis mosseae* and two PGPR strains (*Enterobacter* sp. *and Enterobacter ludwigii)* on tomato tolerance to Cd on a Cd contaminated soil. Plant shoot weight was 39% higher than in the AMF single treatment, and Cd translocation factor was 30% lower. Dhawi et al. (2015) reported an increase of nutrient uptake and growth of *Zea mays* subjected to Cu, Zn and Fe stress, as facilitated by synergistic effects of an AMF mix and a *Pseudomonas* sp. strain. The collaborative interaction between three AMF isolates and four PGPR strains were found to be effective on Fe^3+^ phytoremediation by *Pennisetum glaucum* and *Sorghum bicolor* in a metal contaminated soil (Mishra et al., 2016). In this study, the combination of AMF and bacteria exhibited higher phytoremediation efficiency than AMF treatment alone. Both AMF and PGPR produced siderophores, which explained their additional effects. Barros et al. (2024) showed that co-inoculation of sorghum with *Azospirillum brasilense* and AMF isolate of *Rhizoglomus clarum* in a Cu-contaminated soil was effective in reducing copper concentrations in the shoots to levels below the limits established by Brazilian legislation, with translocation factor value of 0.049. A*. brazilense* increased mycorrhizal colonization by *R. clarum*. The combination of *R. Clarum* and *A. brasilense* improved shoot and root dry biomass compared with R. clarum treatment alone (64.4% and 115.4%, respectively). PGPR and AMF can increase or decrease metal translocation and accumulation in aerial parts, depending on the microbe isolate and plant species (Rajkumar et al., 2009; Amir et al., 2014; Banerjee et al., 2025, Zou et al., 2025), thereby favoring phytoextraction or phytostabilization. The increase in plant biomass due to these microbes also improves phytoremediation potential by increasing storage volume. All the studies clearly demonstrate that AMF and PGPR can develop efficient collaborative interactions and illustrate the important potential of this approach in the phytoremediation of metal contaminated soils, and the restoration of degraded metal mining areas.

**Table 1:** Studies on combined effects of AMF and bacteria in phytoremediation and restoration of metal-stressed soils.

AMF + Bacteria (PGPR/MHB)	Plant species	Metals/site	Main effects	References
*Rhizophagus irregularis* + Synthetic bacterial community	*Glycine max*	As	Enhanced plant biomass, mycorrhizal colonization, and stress tolerance	Khan et al. (2025)
*Rhizoglomus intraradices* + *Pseudomonas fluorescens*	*Solanum lycopersicum*	Cd, Zn	Enhanced nutrient uptake and metal tolerance; mitigated Cd/Zn stress	Zhang et al. (2025)
*Funneliformis mosseae* + bacteria	*Astragalus adsurgens; Stipa grandis*	Cd, in saline soil	Improved growth; reduced Cd and Na in shoots	Jia et al. (2025)
*Acaulospora scrobiculata, Rhizoglomus clarum* + *Azospirillum brasilense*	*Sorghum bicolor*	Cu	Reduced Cu content in shoots	Barros et al. (2024)
Commercial inoculants of AMF + symbiotic N-fixing bacteria	*Pterocarpus indicus*	Ni, Cu, Mo, Mn	Reduced Cu and Mo in soils	Magsayo et al. (2024)
*Funneliformis mosseae* + *Mesorhizobium huakuii*	*Robinia pseudoacacia*	Cd	Increased plant biomass, mycorrhizal colonization, and Cd immobilization, reduced Cd toxicity	Zhang F. et al. (2024)
*Glomus* spp. + *Rhizobium* spp.	*Robinia pseudoacacia*	Cd	increased N fixation, P uptake, and plant biomass, reduced Cd availability	Zou et al. (2025)
*Funneliformis mosseae* + *Bacillus cereus*	*Lygeum* sp*artum*	Multi-metal mine tailings	Increased plant biomass, and enzyme activities, improved phytostabilization	Caravaca et al. (2023)
AMF + bacteria	*Astragalus adsurgens*	Cd, Pb, in saline soil	Alleviated Cd and Pb	Li et al. (2023)
*Rhizophagus intraradices* + *rhizosphere bacteria*	*Acorus calamus*	Cr	AMF increased bacterial diversity and abundance, inducing decrease of Cr in rhizosphere soil;	Wei et al. (2023)
*Claroideoglomus claroideum* + *Meyerozyma guilliermondii, Rhodotorula mucilaginosa*	*Oenothera picensis*	Cu (mine tailings)	Increased plant biomass and antioxidant activity, reduced Cu bioavailability	Pérez et al. (2023)
Rhizophagus *irregularis* + *Cupriavidus* sp.	*Zea mays*	Cd, Zn	Increased anaerobic digestion and biogas production	Paulo et al. (2023)
AMF + indigenous bacteria	*Lolium multiflorum, Zea mays, Medicago sativa*	Zn, Cu	Decreased Zn and Cu in river sediment	Zhang et al. (2022)
*Glomus* sp., *Sclerocystis* sp., *Acaulospora* sp + *Proteus* sp., *Pseudomonas* sp., *Ensifer meliloti*	*Medicago sativa*	Cu, Zn, Pb, Cd	Increased plant biomass, decreased heavy metal concentrations	Raklami et al. (2022a)
*Rhizophagus irregularis, Funneliformis mosseae* + *Pseudomonas fluorescens*	*Cupressus arizonica*	Cd	Reduced Cd translocation and toxicity; increased glomalin in soil	Aalipour et al. (2021)
*Rhizophagus neocaledonicus, Claroideoglomus etunicatum* + *Curtobacterium citreum*	*Tetraria comosa*	Ni (ultramafic soil)	Increased plant biomass, mycorrhizal colonization, and nutrient uptake; reduced metal translocation	Bourles A. et al., Mycorrhiza (2020)
*Funneliformis mosseae* + *Enterobacter* sp. and *Enterobacter ludwigii*	*Lycopersicon esculentum*	Cd	Improved plant growth, and Cd tolerance	Li et al., 2020
*Rhizophagus irregularis* + *Pseudomonas fluorescens*	*Salix alba*	Pb, Cu, Cd	Improved plant growth, mycorrhizal colonization, Pb, Cu, and Cd bioconcentration factor	Mokarram-Kashtiban et al. (2019)
*Claroideoglomus claroideum* + *Pseudomonas libanensis*	*Helianthus annuus*	Ni and salinity	Increased plant growth, chlorophyl content, and stress tolerance	Ma et al. (2019)
*Rhizophagus irregularis* + *two streptomycetes*	Mine polluted soil, no plants added	Heavy metals	Increased functional microbial diversity, microbial activity, and soil organic matter	Schindler et al. (2017)
*Glomus, Acaulospora*, *Scutellospora* + *Streptomyces, Azotobacter, Pseudomonas, Paenibacillus*	*Pennisetum glaucum, Sorghum bicolor*	Fe^3+^	Increased Fe^3+^ absorption	Mishra et al. (2016)
AMF mix + *Pseudomonas* sp.	*Zea mays*	Cu, Zn, Fe	Improved plant growth, and nutrient uptake	Dhawi et al. (2015)
AMF + PGPR	*Eucalyptus camaldulensis*	Heavy metals	Activated photosynthesis, Increased glutathione levels, and heavy metal chelation	Guarino et al. (2014)
*Glomus* spp. + *Bacillus cereus, Candida parapsilosis*	*Trifolium repens*	Al, Mn, Cu Cd, Ni, MO, Zn, As	Increased plant biomass, N, P, K contents, and mycorrhizal colonization, reduced metal concentrations in shoots	Azcón et al. (2010)
*Glomus mosseae* + *Rhizobium trifolii, Brevibacillus brevis*	*Trifolium repens*	Zn	Increased plant biomass, and nodule number, decreased Zn absorption, and Zn toxicity	Vivas et al. (2006)
*Glomus mosseae + Brevibacillus brevis*	*Trifolium repens*	Cd	Improved shoot and root biomass, nodule number, mycorrhizal colonization, reduced Cd in shoots	Vivas et al. (2003)

However, the cooperation between AMF and PGPR in plant growth and adaptation can be limited by some factors. Duan et al. (2025) reported that the early-stage reciprocal cooperation between *Rhizophagus irregularis* and *Rahnella aquatilis*, a phosphate solubilizing bacterium, was reduced when phosphorus was sufficiently available, so that the plant could absorb it directly. Moreover, they showed that the uptake of P by *R. irregularis* was higher in soil with low P, and that the acquisition of carbon by *R. aquatilis* was also better in low P condition, thus revealing an aspect of the cooperation mechanism between the two microbes. Another study (Feng et al., 2023) highlighted the effect of soil pH on the cooperation between AMF and bacteria to facilitate plant growth. This cooperation was significant only when the pH corresponded to the native soil’s pH conditions (4.85), and the positive effect of the co-inoculation was reduced even at a pH value relatively neutral (7.44).

### 3.2 AMF and MHB

MHB have been found associated with AMF spores and mycelium, but can also be inhabitants of the rhizosphere interacting with AMF (Nasslahsen et al., 2022; Williams et al., 2024). Their effects on AM symbiosis occur through different mechanisms, among which: stimulation of the production, survival, and germination of AMF spores, mycelial growth enhancement, stimulation of AM establishment, improvement of mycorrhizal colonization, P solubilization, mineral uptake improvement, promotion of plant growth through hormone production, and enhancement of the plant receptivity to mycorrhizal infection (Lies et al., 2018; Kumar Gupta and Chakraborty, 2020; Williams et al., 2024). MHB are often naturally associated with AMF spores. Spore germination can then be stimulated by their volatile or non-volatile produced compounds (Xavier and Germida, 2003; Lies et al., 2018). They can also erode spore layer, then facilitating spore germination (Roesti et al., 2005). Some MHB are antagonists of plant pathogens and promote AMF development at the same time (Barea et al., 1998; Budi et al., 1999; Sundram et al., 2011). Sundram et al. (2011) reported that *Pseudomonas aeruginosa* and *Bacillus cepacian* are antagonistic to the phytopathogenic fungus *Ganoderma boninense* but, in contrast, promote AMF spore germination and hyphal development. Budi et al. (1999) found that *Paenibacillus* sp. is antagonistic to *Phytophtora parasitica* and improve *Glomus mosseae* development. Three antagonistic peptides produced by *Paenibacillus* sp. have been found effective against several Gram-negative bacteria and soilborne pathogenic fungi (Selim et al., 2005).

The success of AMF partly depends on soil receptivity to these symbionts. The receptivity is defined as the capacity of the soil to maintain AMF population and soil mycorrhizal infectivity (Duponnois et al., 2005). MHB can enhance soil receptivity to AMF (Duponnois and Plenchette, 2003; Singh et al., 2013), increasing the duration of AMF strains in soil after inoculation.

MHB are also effective under metal-stressed conditions. The presence of *Brevibacillus brevis* increases spore germination, presymbiotic fungal growth and mycorrhiza formation under toxic concentrations of metals (Vivas et al., 2005). At Cd concentrations below 20 mg mL-1, the bacteria increased the germination rate of *G. mosseae* spores by 233%. The spore germination rate increased by 180% in the presence of the bacteria under 200 mg mL-1 of Zn. *B. brevis* also had a stimulating effect on the hyphal development by 73% and 154% under 10 and 20 mg Cd mL-1, respectively, and by 65% and 132% under 50 and 200 mg Zn mL-1, respectively, as compared to spores that were not inoculated with bacteria. Azcón et al. (2010) reported that dual inoculation of *Trifolium repens* with *Bacillus cereus* and autochthonous mycorrhizal inoculum composed of *Glomus* species dominated by *G. mosseae* increased shoot biomass by 34% compared to AMF-only treatment and by 84% compared to bacteria-only treatment. AMF root colonization was stimulated by *B. cereus* (37% more). This latter also enhanced nodule production. Al, Mn, Cu, Cd, As, and Ni concentrations in shoots were reduced, but only Al and As were significantly lower than the two single treatments. MHB can have an important effect on mycorrhizal symbiosis by improving mycorrhizal colonization. This effect can be induced by the production by MHB of compounds that stimulate root exudates, resulting in activation of AMF mycelial growth (Lies et al., 2018). Bourles et al. (2020) reported a study on ultramafic soil aiming at restoring mined areas. They worked on *Costularia comosa*, an endemic plant species belonging to Cyperaceae, a family generally considered non-mycorrhizal; except for some New Caledonian Cyperaceae species endemic to ultramafic environments, which are functionally mycorrhizal (Lagrange et al., 2011, 2013), even though mycorrhizal colonization was very low. Aiming to improve the positive effects of the symbiosis, the authors tested a *Rhizophagus neocaledonicus* and *Claroideoglonus etunicatum* inoculum in combination with the bacterial strain BE belonging to *Curtobacterium citreum*, isolated from the rhizosphere of *Costularia* species (Bourles et al., 2020). This strain revealed a positive effect on both AMF spore germination and spore production. The authors showed that mycorrhizal intensity and mycorrhizal frequency increased from 1% and 2.5%, respectively, in the AMF-only treatment, to 7.2% and 33.3% in the AMF+BE treatment. The dry weight of the plants was 2.5 times higher than that of the AMF treatment alone. P, K, and Ca uptake were enhanced in the same proportion. The translocation of Ni, Co, and Cr was reduced by the combined inoculant (94.6%, 153.3%, and 160.0%, respectively); however, this was mainly due to the AMF effect.

In field conditions, as stressed by Lies et al. (2018), the success of the inoculants is related to the soil receptiveness, which is the capacity of the soil to maintain the introduced microbial population. This soil property depends on several factors, such as soil global microbial activity (which can induce more or less competition), the presence of antagonistic taxa, and the compatibility of the inoculum with the soil’s abiotic features (Barea et al., 2005; Lenoir et al., 2016). The combination of AMF with MHB can then enhance the adaptation of the fungal symbionts to soil conditions (Duponnois and Plenchette, 2003) by improving spore germination, mycelial growth, and root colonization.

### 3.3 Phytoremediation mechanisms and applications

As explained by Dong et al. (2024), the mechanisms of phytoremediation mediated by microorganisms involve multiple levels. AMF and PGPR act as interfaces between the soil and the plant, not only by improving plant nutrition and growth but also by alleviating metal toxicity through several mechanisms, favoring metal accumulation in inactive forms within their own structures or in plant organs, inside vacuoles, and bound to cell walls. The specific mechanisms used by AMF and PGPR for neutralizing toxic metals have been explained in the corresponding sections of this paper. Phytoremediation processes facilitated by the combination of AMF and PGPR could correspond mainly to phytoextraction when the interaction between the plant and the inoculum enhances metal accumulation in aerial parts, resulting in TF values higher than 1 (Hnini et al., 2024). It could be more favorable to phytostabilization when this interaction induces metal accumulation preferentially in roots, inducing TF values lower than 1, such as in Mokarram-Kashtiban et al. (2019) study, with Pb and Cu TF < 0.05, and Barros et al. (2024) study, with Pb and Cu TF < 0.05. However, the amounts of metals accumulated in plant organs, neutralized in the root system, or present on the root surface, must be sufficiently high to allow efficient phytoremediation. In this regard, the plant’s ability to absorb and accumulate metals is important. The plant must generally have a certain natural tolerance to these metals. Dong et al. (2024) have listed plant species studied in the context of phytoremediation. Several plant species are metal accumulators or hyperaccumulators and can be used for phytoremediation (Sarma, 2011), but many are not associated with AMF, as is the case with Brassicaceae such as *Alyssum* and *Thlaspi* species.

## 4 Conclusion

In the context of the global ecological crisis, bioremediation and restoration of metal-polluted and degraded ecosystems constitute an important target. The approaches using adapted plant species along with selected microorganisms are of particular interest. The use of AMF and PGPR for restoration and bioremediation of metal-stressed soils is a cost-effective and environmentally sustainable technology, that is likely to become widespread in the coming decades. The combination of these two microbial groups has proven effective due to their synergistic effects resulting from their actions in specific and complementary microbial niches. However, selecting the right plant species and a combination of collaborative microbes having optimal interactions with the plant is necessary.

Further research is needed to deepen our understanding of the synergistic effects between AMF and bacteria, particularly at biochemical and biomolecular levels. It is also important to optimize the selection of AMF and PGPR strains based on their synergistic effects and to develop and improve the formulation of mixed inoculants. Field experiments are also insufficient and need to be developed in the future to confirm laboratory and greenhouse findings, all the more so we know the variability of results in relation to edaphic and climate characteristics, and the low survival rate of inoculants in these conditions. Furthermore, the long-term effects of AMF and bacteria successive inoculations need to be followed-up to precise how these treatments can impact the soil health and its microbial communities. In the near future, we can expect to see an increase in the diversity of microbial inoculants, beyond the AMF-bacteria combination, particularly in relation to omics technologies.
